# Women’s Expectations of and Satisfaction with Antenatal Care Services in a Semi-Urban Setting in Tanzania and Associated Factors: A Cross-Sectional Survey

**DOI:** 10.3390/healthcare11162321

**Published:** 2023-08-17

**Authors:** Rashidi Heri, Khadija I. Yahya-Malima, Mats Malqvist, Lilian Teddy Mselle

**Affiliations:** 1 Department of Nursing Management, Muhimbili University of Health and Allied Sciences, Dar es Salaam P.O. Box 65001, Tanzania; khadija.malima@muhas.ac.tz; 2 Department of Women’s and Children’s Health, Uppsala University, 751 85 Uppsala, Sweden; 3 Department of Clinical Nursing, Muhimbili University of Health and Allied Sciences, Dar es Salaam P.O. Box 65001, Tanzania

**Keywords:** patient satisfactions, patient expectations, antenatal care, Tanzania

## Abstract

Women’s satisfaction has been found to be a good indicator of quality of care and is associated with the utilization of healthcare services. Women’s needs and satisfaction could be improved through the provision of high-quality antenatal care services. This study assessed women’s expectations of and satisfaction with antenatal care and their associated factors in a semiurban setting in Tanzania. A cross-sectional survey using the Expectations and Satisfaction with the Prenatal Care Questionnaire (PESPC) was used to measure pregnant women’s expectations of and satisfaction with antenatal care in the two districts of Kibaha and Bagamoyo, involving 338 pregnant women. The data were analyzed using SPSS version 26. In the expectation subscale, women had high expectations for personalized care (78.4%), other services (from a social worker and nutritionist) (68.8%), and complete care (being taken care of on time, receiving excellent care, and receiving information without prompting) (60.9%), while expectations for continuity of care were the lowest (38.9%). In the satisfaction subscale, women were highly satisfied with providers’ care (being cared for with respect, healthcare provision, the way they were made to feel, and the ability to ask questions) (88.9%), while the least satisfying aspect was system characteristics (e.g., waiting times, scheduling, parking, tests and examinations, and facilities) (63.4%). Distance from a health facility was a significant predictor of both women’s expectations of and satisfaction with antenatal care services, while age and number of pregnancies were also significant predictors of antenatal care expectations. To meet expectations for quality antenatal care services and improve satisfaction with antenatal care, policymakers should improve system characteristics, including the availability of human resources and medical supplies, increased consultation time, flexible schedules, and reduced waiting time. Additionally, ensuring the accessibility of evidence-based health information is important for increasing health literacy among pregnant women.

## 1. Introduction

Antenatal care is designed to assist women in promoting a healthy pregnancy and birth through risk assessments and screenings, as well as health education, counseling, and interventions. Antenatal care (ANC) is the entry point to the healthcare system for most women, especially in developing countries, and it ensures that the health of the mother and fetus is monitored for a healthy pregnancy, safe delivery, and healthy upbringing of the infant. Retaining women in antenatal care is linked to their continued use of other reproductive health services; thus, it is critical to continue improving antenatal care quality.

Despite various efforts to improve the quality of antenatal care, maternal and neonatal mortality rates are still high in Tanzania. The maternal mortality rate was estimated to be 386 per 100,000 live births in the 2015–2016 Tanzania Demographic and Health Survey and Malaria Indicator Survey, which is higher than the global average (216 deaths) and significantly higher than the rate in developed countries, e.g., the United States, which was 14 deaths [1,2,3]. This is happening even though 98% of pregnant women had at least one ANC contact and 51% had the four recommended contacts during pregnancy. Additionally, 64% of women had assisted deliveries in health facilities [1,2]. Understanding women’s needs and expectations for antenatal care will foster the provision of quality care by applying strategies that adequately address their needs. Meeting women’s expectations for antenatal care may increase women’s satisfaction with care and enhance its use and continuity.

The term “expectations” refers to ideal, expected, or desired healthcare to be provided [4]. Expectations are a pregnant woman’s thoughts about what kind of care she will receive and how good it will be [5]. Various factors have been identified that influence pregnant women’s expectations for prenatal care, including their knowledge, exposure, and experience with antenatal care, as well as their expectations for the care provided by their healthcare provider [6]. One good predictor of women’s satisfaction with antenatal care is their expectations for care, so it is crucial to understand what those expectations are and align care strategies to their needs.

Women’s satisfaction with care is a complex and challenging concept to define, as women’s expectations and experiences with care vary. It may be defined as a “positive evaluation of distinct dimensions of pregnancy and childbirth”, or the extent to which the patient’s expectations or care needs are met [4,7]. There is no universally accepted standard measure of antenatal care satisfaction, and satisfaction with care is determined by several factors [4]. These factors cut across structure (appropriate human resources, medications, and supplies), process (interpersonal behaviors, privacy, promptness, cognitive care, perceived competency of the provider, and emotional support), and outcome of care (mother’s and newborn’s health status) [8]. Other factors associated with patient satisfaction include age, gender, level of education, waiting time, doctors’ communication behavior, patient trust level, staff confidence and competence, type of health center, type of pregnancy, access to care, cost, socioeconomic status, and reproductive history [4,9,10]. It is important to explore those factors so that we can incorporate them into care planning.

The desire to satisfy customers is centered on enhancing the care process and the care environment. This needs systemic improvements in healthcare, including technical, interpersonal, and institutional reforms [11,12]. Clients always think about the care they receive, the process, and the environment in which they receive care, and these things all play a role in how satisfied they are. Satisfaction with care has been linked to improved health outcomes, use, adherence to care and treatment, continued use of health services, and a more positive relationship with providers [7,12]. Feedback from women regarding their satisfaction with antenatal care can be used by healthcare providers, hospital administrators, and policymakers to improve maternal services and allocate resources appropriately [13,14]. Patient satisfaction is a common outcome measure for quality of care evaluations [10,13]. It is a particularly important indicator of the quality of care, so it should be measured periodically to inform policymakers of the customer’s perspective on the care provided.

A review of the literature to determine the factors influencing maternal health satisfaction in developing countries revealed that maternal satisfaction was generally high across developing countries [8]. In the 24 studies reviewed by Srivastava et al. (2015), over 75% of women rated their care as satisfactory. Additionally, the proportion varied between 50% and 75% in ten studies, while it was less than 50% in only three [8]. According to a recent study conducted in Ethiopia, 74% of mothers were satisfied with the antenatal care services provided by public health institutions in Hosanna Town [15]. Some studies conducted in Tanzania using the SERVQUAL questionnaire in two district hospitals revealed widespread dissatisfaction with healthcare quality [16] and with the quality of care received in reproductive and child health clinics [17]. Little has been documented about overall expectations of and satisfaction with antenatal care in Tanzania and their predictors, so the issue needs more exploration.

One of the most reliable ways to predict patient satisfaction with care is through expectations of care, which have a positive correlation with satisfaction, according to various studies [7]. On the contrary, Roder-DeWan et al. (2019) hypothesized that people with low expectations of care are more likely to be satisfied with even substandard care [18]. The complex relationship between how people evaluate care and being satisfied with it requires good measuring tools for both expectations of and satisfaction with care, and a cautious interpretation of the results is needed. Various tools are used to measure expectations of and satisfaction with care, and one of them is the Expectations and Satisfaction with Prenatal Care Questionnaire (PESPC), which is used in this study. While some studies in Tanzania have explored women’s experiences and satisfaction with antenatal care services, these studies did not report the expectations of and satisfaction of women in semi-urban settings in Tanzania. Therefore, this study aims to fill this knowledge gap by examining the expectations and satisfaction levels of women receiving antenatal care in a semi-urban area of Tanzania. By understanding the level of women’s expectations of and satisfactions with antenatal care and the factors associated with it, healthcare providers will be able to tailor services to better meet the needs of pregnant women in this specific context. The purpose of this study was therefore to investigate the satisfaction and expectation levels of pregnant women with the antenatal care provided in midlevel health facilities in semi-urban Tanzania.

## 2. Materials and Methods

### 2.1. Study Design and Setting

A cross-sectional survey was conducted among pregnant women in the Tanzanian districts of Kibaha and Bagamoyo. This study was nested in a larger survey that aimed to determine the effectiveness of a group antenatal care model on quality and satisfaction with antenatal care in a semi-urban setting in Tanzania. The survey was conducted in Kibaha and Bagamoyo, which have a total population of 594,205 (290,202 males and 304,003 females) [19]. Study participants were pregnant women attending the selected health facilities with a gestational age of 20 to 26 weeks. The gestational age was chosen to allow for the timely recruitment of eligible women of the same gestational age for group antenatal care, which requires pregnant women to be followed together throughout the pregnancy period. A total of 338 pregnant women were involved in the study. The ethical clearance for the study was granted by the Muhimbili University of Health and Allied Sciences. All participants were informed about the purpose of the study and signed the consent form to be involved in it.

### 2.2. Sample Size Estimation and Participants Recruitment

The sample size was estimated using Epi Info 7 statistical software using a power of 80%, a two-sided confidence level of 95%, and a ratio of unexposed and exposed of 1. The expected satisfaction changes from good to very good (75% to 95%) were estimated based on a study conducted in Iran [20]. The dropout rate of 15% was added to the sample size of 136 women. Pregnant women attending the antenatal clinics at the Mkoani, Mlandizi, and Kerege health centers and Bagamoyo district hospital were invited to participate in the study. To be included in the study, women had to have a pregnancy without medical, obstetrical, or surgical complications and a gestation age of between 20 and 26, have made no more than three ANC contacts, and speak Kiswahili [21]. The gestational age restriction aimed to facilitate the early recruitment of eligible women for group antenatal care intervention, which requires pregnant women to be cared for in a group throughout their pregnancy. The number of women involved in each health facility was 80 from Mkoani, 84 from Mlandizi, 90 from Kerege Health Centre, and 84 from Bagamoyo District Hospital. This study was part of a larger intervention study of group antenatal care. Less than three antenatal contacts may lower women’s exposure to standard antenatal care which was used as a control group vs. group antenatal care intervention. The researchers included four qualified midwives, one in each clinic. The researchers were trained on how to administer the questionnaires. They invited women to take part in the study during their routine antenatal visit, and for those who agreed, the questionnaires were administered to each woman via interview.

### 2.3. Questionnaires/Measures

The social demographic information was collected using a questionnaire that included the following factors: age, parity, education level, marital status, financial situation, tobacco use, previous pregnancy complications, and if the pregnancy was planned or not. Additionally, it included biomedical measures such as weight, height, blood pressure, and hemoglobin level. Patient expectations and satisfaction were measured using the Patient Expectations and Satisfaction with the Prenatal Care Questionnaire (PESPC), which is a 41-item tool developed by Omar et al., in 2001 in the USA and aimed to develop a valid means of measuring pregnant women’s satisfaction with prenatal care. The tool was tested by Omar et al., using structural equation modeling and confirmatory factor analysis and was found to be structurally valid with an acceptable level of internal consistency [5]. The tool was also validated in Brazil in 2013 by Prudêncio et al., and found to have acceptable internal consistency (Cronbach’s alpha ≥ 0.70), a strong correlation in test–retest (r = 0.82; *p* < 0.001) for the domain expectations, and a moderate correlation (r = 0.66; *p* < 0.001) for the satisfaction domain [22].

The study tools were translated into Kiswahili and back translated to English to check for consistency between the two versions. One of the 41 items on the original instrument was removed after it was translated into Kiswahili. We chose not to include the item “I am satisfied with the services of a public health nurse as part of antenatal care” because the few public health nurses available in Tanzania work in the antenatal care clinic as nurses. After the back translation, there was not much difference between the two documents, so the Kiswahili version was chosen.

The final tool had 40 items with two domains, expectations and satisfaction. Each domain had four subscales: complete care, provider continuity, personalized care, and the availability of other services for the expectations domain; and information, provider care, staff interest, and system characteristics for the satisfaction domain. The items were rated on a Likert scale of 1 (strongly disagree), 2 (moderately disagree), 3 (slightly disagree), 4 (slightly agree), 5 (agree), and 6 (strongly agree). A high score on the scale means that the person has high expectations and a high satisfaction level. The final instrument had a high level of internal consistency with an overall Cronbach’s of 0.87; 0.752 for the expectations domain, and 0.878 for the satisfaction domain, as shown in Table 1. The expectation scale was also moderately positively correlated to the satisfaction scale, (0.376; *p* < 0.001).

Four dimensions of expectations were measured using the expectations subscales: expectations for complete care (being taken care of on time, receiving excellent care, and receiving information without prompting), provider continuity (being cared for by the same doctor or midwife from ANC clinic to delivery), personalized care (care about their mental and physical state, gentle listening and care, and appropriate referrals), and other services (services from a social worker and nutritionist).

Four dimensions of satisfaction were measured using the satisfaction subscales: satisfaction with information (e.g., explanations of care and preparation for labor and delivery), satisfaction with provider care (e.g., respect, care provision, the way they were made to feel, ability to ask questions), satisfaction with staff interest (e.g., interest and concern shown by staff and time spent for care), and satisfaction with system characteristics (e.g., waiting times, scheduling, parking, tests and examinations, and facilities).

### 2.4. Data Analysis

The statistical package SPSS-26 was used to analyze the data. The normality of the distributions of all responses was determined using the Kolmogorov–Smirnova test to determine whether parametric or non-parametric analyses were appropriate, and predominantly non-parametric statistics were used. All variables were subjected to descriptive analysis. The data were described by frequency and percentage, mean and standard deviation, and median. The scores for the domains of expectations and satisfaction, as well as their respective subscales, were added. The total of each domain and subscale was divided by the number of items in that domain or subscale to obtain an average score. The average score was divided by 6 and multiplied by 100 to obtain the percentage score. To obtain a binary score for either low or high satisfaction or expectations, a cutoff point was selected for those scoring an average of 1 to 3.999 (I disagree strongly to I disagree slightly) as disagreeing with satisfaction and expectation statements (low score) and those scoring an average of 4 to 6 (I agree slightly to I agree strongly) as agreeing with satisfaction and expectation statements (high score). The binary analysis of factors associated with expectations and satisfaction was conducted using chi-square. Those factors with *p* ≤ 0.25 were included in the logistic regression model [23], including those factors supported by the literature to be associated with satisfaction and expectations of ANC care. The binary logistic regression technique using enter variable selection was used to determine the factors that influenced high or low levels of satisfaction and expectations with care. Multi-collinearity among the explanatory variables was checked using the variance inflation factor (VIF value ≤ 2.0 indicates the absence of multicollinearity). The stability of the model was checked using the bootstrap method (1000 samples). The significance level was set at 0.05.

## 3. Results

### 3.1. Demographic Information

The average age of the women was 26.3 ± 5.9 years, their average weight was 63.8 ± 12.4 kg, and their average height was 156.3 ± 6.1. Most of the women (82.2 percent, (*n* = 278) were taller than 150 cm, which is the cut-off for the risk of spontaneous vaginal delivery failing. In total, 88.1 percent of the respondents (*n* = 290) were either cohabiting or married. In terms of education, 56.5 percent of the women (*n* = 191) had finished primary school, and 34.6 percent of the respondents (*n* = 117) had secondary education or higher. The primary source of income for 55.9 percent (*n* = 189) of the women was small business and agriculture, and only 34.6 percent (*n* = 117) reported that their family income was sufficient to meet their family’s needs. Around three quarters (71%, *n* = 240) of the women reported living far from an ANC clinic. A third of the women (31.1%, *n* = 105) were primigravida, and a third (32%, *n* = 105) had unintended pregnancies. Table 2 provides additional details.

### 3.2. Health Financing, Decision Making, and Risk Behaviors

The majority of women, 94.4% (*n* = 319), had no health insurance, and only 6.5% (*n* = 22) of the other women paid for their ANC visit. The decision for the ANC visits was made to a large extent by both women and partners (73.4% (*n* = 248)), with fewer by women (17.8% (*n* = 60)), and the least by partners (8.9% (*n* = 30)). Cigarette smoking was extremely low; only 0.3% (*n* = 1) reported that they were smokers and 6.2% (*n* = 21) were living with a smoker. Alcohol consumption was also very low; only 1.8% (*n* = 6) reported they drank alcohol during pregnancy and 2.4% (*n* = 8) reported they drank but not during pregnancy.

### 3.3. Pregnant Women’s Expectations of and Satisfaction with Antenatal Care

The overall mean and median (Md) percentages for the expectation scale were 64.4% (Md = 65.9%). The highest average score for the expectation subscale was obtained for personalized care at 78.4% (Md = 83.3%), and the lowest average score was obtained for other services at 39.0% (Md = 25.0%). The average scores for complete care and other services were 60.9% (Md = 62.5%) and 68.3% (Md = 75.0%), respectively. After categorizing the level of expectations as high or low, half of the women, 169 (50%), had a high level of expectations. Table 3 provides additional information.

The overall satisfaction scale score was 74.9% (Md = 75.9%). The highest average score was obtained for provider care at 86.3% (Md = 88.9%), and the lowest average score was obtained for other services at 63.5% (Md = 65.0%). The average scores for information and staff interest were 81.4% (Md = 86.1%) and 75.8% (Md = 75.0%), respectively. After categorizing the level of satisfaction as high or low, the majority of women, 253 (74.9%), had a high level of satisfaction. Table 3 provides additional information.

### 3.4. Factors Associated with Pregnant Women’s Expectations of and Satisfaction with Antenatal Care

A chi-square test for independence was conducted involving factors in the demographic information (age, marital status, education, occupation, perceived level of income, distance from a health facility, number of pregnancies, planned pregnancy, decision for an ANC visit, and cost payment) to determine factors associated with women’s overall expectations of and satisfaction with antenatal care. Regarding women’s overall expectations of antenatal care, one factor (perceived distance to the antenatal clinic) was significantly associated with it (X^2^ (1, *n* = 338) = 8.278; *p* = 0.004). Regarding women’s overall satisfaction with antenatal care, two factors were significantly associated with it: the perceived distance to the antenatal clinic (X^2^ (1, *n* = 338) = 13.616, *p* ≤ 0.001) and making decisions about attending an antenatal care clinic (X^2^ (1, *n* = 338) = 12.046, *p* = 0.002).

### 3.5. Factors Affecting Expectations for and Satisfaction with Antenatal Care (ANC) in a Semi-Urban Setting in Tanzania

The logistic regression was conducted to assess the influence of a range of factors on pregnant women’s expectations for and satisfaction with ANC care. The model included nine independent variables, those that achieved *p* ≤ 0.25 during univariate analysis [23] and those supported by the literature to be associated with expectations of and satisfaction with antenatal care. Three of the independent variables were identified as predictors of expectations for antenatal care: age, number of pregnancies, and perceived distance from the health facility. The odd ratio of 3.625 for the number of pregnancies suggests that women with four pregnancies or more were 3.6 times more likely to have high expectations for ANC care than those in their first pregnancy. The odd ratio of 2.159 for distance from health facilities suggests that women who lived far from an ANC clinic were 2.2 times more likely to have high expectations for ANC care than those who lived nearby. Additionally, women who were 35 years old or older were 0.37 times less likely to have high expectations for ANC care than women who were 24 years old or younger.

Regarding factors affecting satisfaction with antenatal care, only one independent variable was identified as a predictor of satisfaction with antenatal care (distance from a health facility). The odd ratio of 2.576 for distance from health facilities suggests that women who lived far from an ANC clinic were 2.6 times more likely to be satisfied with ANC care than those who lived nearby. Even after bootstrapping 1000 samples, the two models remained unchanged, showing that they are stable. Table 4 provides additional information.

## 4. Discussion

The purpose of this study was to determine whether pregnant women in Tanzania were satisfied with the prenatal care they received at midlevel health facilities, whether it met their needs, and what factors contributed to the expectations of and satisfaction with ANC care. The results showed that women were highly satisfied with antenatal care, while their expectations were slightly lower but positively correlated with their satisfaction. The satisfaction ratings measured with the same instrument were higher than those previously reported in the USA [5] but lower than those reported in Belgium [4]. However, the satisfaction levels reported in Tanzania using other instruments showed low satisfaction with antenatal care and healthcare [16,17]. However, most studies from developing countries found high satisfaction with antenatal care [8,10]. The main thing that indicates the provision of quality of care is how well women’s expectations or needs are met, which is related to how satisfied they are with care. In a Canadian study [24], the quality of prenatal care and the interpersonal style of the provider were found to account for 80% of the variance in overall satisfaction. Women’s satisfaction and utilization of prenatal care may be improved by enhancing the quality of care, the interpersonal style of providers, and involving women in decision making about their care, as well as by enhancing the structural characteristics of antenatal care.

Expectations associated with different aspects of antenatal care were measured using four subscales: complete care, provider continuity, personalized care, and other services. Women had the highest expectations for personalized care (care about the mental and physical state, gentle listening and care, and appropriate referrals), followed by other services (services from a social worker and nutritionist). Women expected to be gently cared for, listened to, and have their mental and physical feelings recognized and referred to as needed. They were also highly expected to receive more services, such as social aspects of care from social workers and nutrition care from nutritionists. Women knew how important these components of care were to their health, so the health system had to deal with them if it wanted to keep its customers satisfied. The average expectations were reported for complete care (being taken care of on time, receiving excellent care, and receiving information without prompting), and the lowest scores were reported for provider continuity (being cared for by the same doctor or midwife from the ANC clinic to delivery) (Supplementary File S1). This low score could be because they have had bad experiences with the health system not taking care of these things well or because they did not know how important these things are. Therefore, there is a need to improve the continuity of care, information delivery, and time management in antenatal clinics.

Satisfaction with different aspects of antenatal care was measured using four subscales: information, provider care, staff interest, and system characteristics. On the satisfaction subscale of provider care (e.g., respect, care provision, the way they were made to feel, ability to ask questions), women were highly satisfied with this aspect of antenatal care. The staff interest subscales (e.g., interest and concern shown by staff and time spent on care) for women were also highly satisfied, except for the item on the time staff spent talking about issues of women’s interest, which received the lowest satisfaction scores (Supplementary File S2). Women were less satisfied with the information provision (e.g., explanations of care and preparation for labor and delivery), especially with information about parenting a newborn and preparation for labor and delivery (Supplementary File S2). The subscale system characteristics (e.g., wait times, scheduling, parking, tests and examinations, and facilities) received the lowest scores, particularly the items’ ability to schedule antenatal visits at a convenient time and ease of rescheduling the visits. Additionally, clinic facilities such as examination rooms, waiting rooms, and availability of recommended tests had the lowest scores (see Supplementary File S2).

The satisfaction results were similar to those reported in Belgium [4] but different from those reported in the USA, where the scores were lower across the four subscales [5]. This may be due to their level of expectation, as US women have higher expectations compared to satisfaction levels, while in Tanzania and Belgium, women had lower expectations than satisfaction. Patients with unreasonably high expectations may be dissatisfied if their expectations are not met, whereas women with low expectations may be satisfied with the care that meets their needs [5]. Women appear satisfied with the care provision aspects of antenatal care, but the provision of information and system characteristics are not appreciated by women. Efforts should therefore be increased to enhance the sharing of vital information during antenatal contacts and to explore and address their expectations. Additionally, there is a need for more improvement in staffing and facilities, such as laboratory equipment and test kits, clinic environments, and building space. This area for quality improvements (waiting times, the quality of basic amenities, and communication with healthcare providers) has also been identified elsewhere as a priority for quality improvement [11].

Many studies have been conducted to find out how different factors directly affect patient perceptions of care or patient satisfaction with care, but patient expectations are not explored further. The risk factors for low expectations of antenatal care were found to be fewer than four pregnancies, living near health facilities, and women who were under 35 years of age. The reason for this finding may be that expectations are determined by experience, level of knowledge, and cultural norms, so women with more pregnancies know what to expect from care and are more likely to have high expectations. Additionally, people living far from their clinic have made a careful choice to select an appropriate clinic that they think will provide better care, so they have realistic expectations for it. In the case of young women, those under 35 years of age may be less experienced and thus more likely to have low expectations for antenatal care. Younger age was also found to be a risk factor for low expectations in Belgium. In Belgium, factors such as high education and low income were found to be risk factors for low expectations, but in Tanzania, they were not [4]. Age, being married, and reporting intimate partner violence were also risk factors for low expectations [4]. There is a need to explore women’s expectations of antenatal care so that care can be tailored to meet the needs of all women.

In this study, demographic characteristics such as education, perceived level of income, age, marital status, and occupation were not significantly associated with satisfaction with antenatal care. A study conducted in Belgium using the same tool also found that education and level of income were not associated with satisfaction with antenatal care [4]. In our study, decision making about attending antenatal care and distance from health facilities were significantly associated with satisfaction with antenatal care. There is no consensus on factors associated with satisfaction with antenatal care in the literature, but the following factors have been reported in many works in the literature to be significantly associated with a client’s satisfaction with antenatal care; education, level of income, type of pregnancy, history of stillbirth, age, gender, waiting time, doctors’ communication behavior, patient trust level, employment, residence, privacy, cleanness, distance from health facilities, respect, good physical environment, availability of adequate human resources, medicines, and supplies, interpersonal behavior, privacy, promptness, cognitive care, perceived provider competency, emotional wellbeing, ANC visits, gestational age, and spending money out of pocket [8,10,18,25,26]. Satisfaction with antenatal care is a complex phenomenon and is affected by a lot of factors, so careful consideration is required in the planning and implementation of care strategies to satisfy as many women as possible, thus ensuring that they continue to use healthcare services.

### Strengths and Limitations

The Expectations and Satisfaction with Prenatal Care Questionnaire (PESPC), which is a valid and reliable tool, was used to measure expectations of and satisfaction with antenatal care, as well as their predictors. This is one of the study’s strengths. Another strength was the ability to survey women before they gave birth, thereby removing the potential bias of childbirth. Face-to-face recruitment and data collection are known sources of selection and response bias, which was reduced by keeping healthcare workers from antenatal clinics out of the selection process for study participants. The study was conducted in a semi-urban area in the coast region of Tanzania; therefore, the results may be generalized to similar settings.

## 5. Conclusions

This study identifies the components where women in Tanzania have high and low expectations, those with which they have high and low satisfaction, and the factors associated with them. Women expected to be gently cared for, listened to, and have their mental and physical feelings recognized and referred to as needed. They also expected to receive more services, such as social aspects of care from social workers and nutrition care from nutritionists. They had low expectations of being taken care of on time, receiving excellent care, receiving information without prompting, and having provider continuity. This was reflected in their satisfaction, where women were less satisfied with the information provision, especially with information about parenting a newborn and preparation for labor and delivery system characteristics, their ability to schedule antenatal visits at a convenient time, and ease of rescheduling the visits, the clinic facilities, examination rooms, waiting room, and availability of recommended tests. This study provides insights into women’s expectations of and satisfaction with antenatal care services in a semi-urban setting in Tanzania. It underscores the need for targeted interventions aiming to improve care and meet the needs of pregnant women in Tanzania. Addressing the factors associated with satisfaction may contribute to improved maternal and child health outcomes and promote overall well-being in the community. The further implementation of evidence-based strategies may ensure optimal antenatal care experiences for women in similar settings. Efforts should therefore be made to enhance the sharing of health information during antenatal contacts, improve staffing levels, ensure continuity of care, and increase laboratory equipment, test kits, and infrastructure. Studies predicting expectations of and satisfaction with care are required to improve care planning and implementation strategies to promote the use of healthcare services.

## Figures and Tables

**Table 1:** Reliability of the expectations and satisfaction with the prenatal care questionnaire (PESPC).

Subscale	Cronbach’s Alpha	Cronbach’s Alpha on Standardized Items
Total PESP Tool	0.879	0.889
Expectation Scale	0.752	0.756
Complete Care	0.192	0.20
Provider Continuity	0.635	0.638
Personalized Care	0.723	0.728
Other Services	0.734	0.735
Satisfaction Scale	0.878	0.889
Information	0.664	0.684
Provider Care	0.754	0.757
Staff Interest	0.755	0.786
System Characteristics	0.819	0.809

**Table 2:** Social demographic characteristics of pregnant women (*n* = 338).

Variables	*n* (%)
**Age in years (*n* = 338)**	
≤24 Years	156 (45.6)
25–34 Years	144 (42.6)
≥35 Years	40 (11.8)
Mean (SD) Years	26.3(5.9)
**Height in centimeters (*n* = 338)**	
Height of the mother, ≤150 cm	60 (17.8)
Height of the mother, >150 cm	278 (82.2)
Mean (SD) cm	156.3(6.1)
**Marital status (*n* = 338)**	
Married	230 (68.0)
Single	40 (11.8)
Living together (cohabiting)	68 (20.1)
**Level of education (*n* = 338)**	
No formal education	30 (8.9)
Primary education	191 (56.6)
Secondary education and higher	117 (34.6)
**Current occupation (*n* = 338)**	
Wage employment	18 (5.3)
Business	173 (51.2)
Farming and others	147 (43.5)
**Perceived level of income to meet family needs (*n* = 338)**	
Enough	116 (34.3)
Inadequate	222 (65.7)
**Distance from a health facility (*n* = 338)**	
Near	98 (29.0)
Far	240 (71.0)
**Number of pregnancies (*n* = 338)**	
1	105 (31.1)
2 and 3	157 (46.4)
≥4	76 (22.5)
**Planned pregnancy (*n* = 338)**	
Yes	230 (68)
No	108 (32)

**Table 3:** Descriptive statistics scales and subscales: Expectation and Satisfaction with Antenatal Care *n* = 338.

Scales and Subscales	Number of Items	Score Possible Interval	Score Average (SD)	Median Score	Score Percentage	Median Percentage	Categorized Score (Low and High Expectation and Satisfaction Level *n* (%)	
							Low	High
**Expectations**	**12**	**12–72**	**46.37 (11.72)**	**47.5**	**64.40**	**65.9**	**169 (50.0)**	**169 (50.0)**
Complete care	4	4–24	14.62 (4.11)	15.0	60.92	62.5	199 (59.9)	139 (41.1)
Provider continuity	2	2–12	4.67 (3.25)	3.0	38.95	25.0	271 (80.2)	67 (19.8)
Personalized care	4	4–24	18.81 (5.18)	20.0	78.37	83.3	74 (21.9)	264 (78.1)
Other services	2	2–12	8.26 (3.73)	9.0	68.83	75.0	148 (43.8)	190 (56.2)
**Satisfaction**	**28**	**28–168**	**125.75 (21.64)**	**127.5**	**74.85**	**75.89**	**85 (25.1)**	**253 (74.9)**
Information	6	6–36	27.28 (5.85)	27.0	75.78	75.00	78 (23.1)	260 (76.9)
Provider care	6	6–36	31.08 (4.83)	32.0	86.34	88.89	28 (8.3)	310 (91.7)
Staff interest	6	6–36	29.31 (5.85)	31.0	81.41	86.11	48 (14.2)	290 (85.8)
System characteristics	10	10–60	38.07 (11.48)	39.0	63.46	65.00	170 (50.3)	168 (49.7)

**Table 4:** Binary logistic regression analysis showing factors affecting expectations for and satisfaction with antenatal care (ANC) in a semi-urban setting in Tanzania (*n* = 338).

Characteristics	Low Expectations vs. High Expectations	Low Satisfaction vs. High Satisfaction
**Overall Expectations level**	**95% C. I for Odds Ratio**	**95% C. I for Odds Ratio**
**Age in years**		
≤24 Years	Reference	Reference
25–34 Years	0.71 (0.40–1.23)	0.75 (0.39–1.43)
≥35 Years	0.37 (0.14–0.94) *	1.25 (0.41–3.80)
**Level of education**		
No formal education	Reference	Reference
Primary education	1.41 (0.60–3.35)	0.99 (0.36–2.70)
Secondary education and higher	1.63 (0.63–4.20)	0.99 (0.33–2.95)
**Current occupation**		
Wage employment	Reference	Reference
Business	0.61 (0.21–1.73)	1.47 (0.48–4.54)
Farming and others	0.41 (0.14–1.18)	1.56 (0.50–4.89)
**Perceived level of income to meet family needs**		
Enough	Reference	Reference
Inadequate	0.88 (0.52–1.46)	1.11 (0.62–2.00)
**Perceived distance from a health facility**		
Near	Reference	Reference
Far	2.15 (1.27–3.65) **	2.57 (1.46–4.53) **
**Number of pregnancies**		
1	Reference	Reference
2 and 3	1.38 (0.77–2.47)	1.12 (0.57–2.20)
≥4	3.62 (1.56–8.42) **	1.35 (0.51–3.54)
**Planned pregnancy**		
Yes	Reference	Reference
No	0.69 (0.41–1.13)	0.59 (0.33–1.05)
**Decisions for attending antenatal care clinic**		
Myself	Reference	Reference
My husband/partner/others	1.25 (0.49–3.22)	0.41 (0.15–1.08)
Together with my husband/partner	1.13 (0.58–2.20)	1.23 (0.59–2.56)
**Payment for antenatal care clinic**		
Myself	Reference	Reference
My husband/partner/other	1.59 (0.59–4.26)	0.50 (0.14–1.71)

* *p* < 0.05, ** *p* < 0.01.

## Data Availability

Datasets used and/or analyzed during the current study are available from the first author on request.

## Supplementary Materials

The following supporting information can be downloaded at: https://www.mdpi.com/article/10.3390/healthcare11162321/s1.
