# Supplementary material for: Women’s Expectations of and Satisfaction with Antenatal Care Services in a Semi-Urban Setting in Tanzania and Associated Factors: A Cross-Sectional Survey
Source: Healthcare (Basel). 2023 Aug 17;11(16):2321. doi: 10.3390/healthcare11162321 (PMC10454190; doi:10.3390/healthcare11162321)
Supplement: Supplementary file 1 [file healthcare-11-02321-s001.zip › Heri Rashidi Additional File 1.pdf]

Additional File S1: Expectations with Antenatal Care Items Scores Mean, Standard deviation and Median

Expectations Scale	Mean	SD	Median	Subscale
I expected to be seen sooner for my first prenatal visit	4.77	1.762	6	Complete Care
I expected to have my prenatal visits take a long time	2.88	1.887	2	
I expected to get more from my prenatal visits than being weighed and having my baby's heart checked	4.62	1.876	6	
I expected to receive information during my visits without having to ask so many questions	2.30	1.742	2	
I expected to have one provider (=gynaecologist, midwife or doctor) that I routinely see for my prenatal visits	2.24	1.783	1	Provider continuity
I expected to have the provider that I routinely see deliver my baby	2.44	2.013	1	
I expected my provider to care how I feel mentally as well as physically	4.51	1.841	5	Personalized Care
I expected my provider to be gentle during my physical exam	5.08	1.519	6	
I expected someone to listen to my problems	4.75	1.750	6	
I expected a referral when I tell the clinic/office staff about a problem	4.47	1.882	5	
I expected the services of a social worker to be part of prenatal care	3.82	2.152	5	Other services
I expected the services of a nutritionist to be part of prenatal care	4.44	2.054	6	
