# Supplementary material for: Women’s Expectations of and Satisfaction with Antenatal Care Services in a Semi-Urban Setting in Tanzania and Associated Factors: A Cross-Sectional Survey
Source: Healthcare (Basel). 2023 Aug 17;11(16):2321. doi: 10.3390/healthcare11162321 (PMC10454190; doi:10.3390/healthcare11162321)
Supplement: Supplementary file 1 [file healthcare-11-02321-s001.zip › Heri Rashidi Additional File 2 Description.pdf]

Satisfaction with Antenatal Care Domain

The addition file 2 contains a summary of the item scores used to assess the four subscales of satisfaction domain of the Patient Expectations and Satisfaction with Prenatal Care Questionnaire (PESPC). Each item consists of the score mean, standard deviation, and median.
