# Supplementary material for: Women’s Expectations of and Satisfaction with Antenatal Care Services in a Semi-Urban Setting in Tanzania and Associated Factors: A Cross-Sectional Survey
Source: Healthcare (Basel). 2023 Aug 17;11(16):2321. doi: 10.3390/healthcare11162321 (PMC10454190; doi:10.3390/healthcare11162321)
Supplement: Supplementary file 1 [file healthcare-11-02321-s001.zip › Heri Rashidi Additional File 2.pdf]

Additional File S2: Satisfaction with Antenatal Care Items Scores Mean, Standard deviation and Median

Satisfactions Scale	Mean	SD	Median	Subscale
I am satisfied with the explanation my provider gave to me of what was going to happen during my prenatal visits	5.34	1.314	6	Information
I am satisfied with the explanation my provider gave to me about medical procedures	5.51	.990	6	
I am satisfied with the information my provider gave to me about how things are going with my pregnancy	5.51	1.087	6	
I am satisfied with the kinds of things my provider discussed during my prenatal visits	5.11	1.469	6	
I am satisfied with the explanation my provider gave to me about what I can expect about parenting a new- born	2.65	2.073	1	
I am satisfied with the way my provider has prepared me for labour and delivery	3.16	2.237	2	
I am satisfied with the way my provider treats me	5.39	.975	6	Provider care
I am satisfied with the respect that I am shown by my provider	5.39	1.063	6	
I am satisfied with the quality of care that I receive from my provider	5.40	.945	6	
I am satisfied with the way I am made to feel that I am not wasting my provider's time	5.15	1.278	6	
I am satisfied with being able to ask questions without embarrassment	5.05	1.421	6	
I am satisfied with not having to repeat my story every time I come in for a visit	4.70	1.566	5	
I am satisfied with the way the staff expresses concern about my overall personal situation	5.36	1.129	6	Staff Interest
I am satisfied with the time the staff spends talking about things of interest to me	3.70	2.020	4	
I am satisfied with the way the staff treats me	5.27	1.161	6	
I am satisfied with the time the staff takes with me even though I do not have problems with this pregnancy	5.09	1.410	6	

I am satisfied with the interest and concern the staff has shown me	5.08	1.318	6	
I am satisfied with the way the staff deals with all my medical problems	4.81	1.509	5	
I am satisfied with the amount of time I wait to be seen by my provider	3.86	1.996	5	System characteristic
I am satisfied with the total amount of time I spend at the office/clinic	3.88	2.007	5	
I am satisfied with the parking facilities of the office/clinic	3.99	2.001	5	
I am satisfied with the waiting room facilities of the office/clinic	3.67	2.163	4	
I am satisfied with the examination room of the office/clinic	3.74	2.170	5	
I am satisfied with my ability to schedule prenatal visits at a time convenient for me	2.37	1.846	1	
I am satisfied with how easy it is to reschedule my prenatal visits	2.13	1.629	1	
I am satisfied with how easy it was to get prenatal care early in my pregnancy (that is, before the fourth month)	5.49	1.236	6	
I am satisfied with having all the recommended tests	3.54	2.125	4	
I am satisfied with the number of prenatal visits I made until now	5.40	1.066	6	
